# Citrulline prevents age-related LTP decline in old rats

**DOI:** 10.1038/s41598-019-56598-2

**Published:** 2019-12-27

**Authors:** Antonin Ginguay, Anne Regazzetti, Olivier Laprevote, Christophe Moinard, Jean-Pascal De Bandt, Luc Cynober, Jean-Marie Billard, Bernadette Allinquant, Patrick Dutar

**Affiliations:** 1 Centre de Psychiatrie et Neurosciences, UMR_S894, INSERM, Université Paris Descartes, Sorbonne Paris Cité, Paris, France; 2 Service de Biochimie, Hôpitaux Universitaires Paris-Centre, Hôpital Cochin, AP-HP, Paris, France; 3 EA4466 Laboratoire de Biologie de la Nutrition, Faculté de Pharmacie de Paris, Université Paris Descartes, Sorbonne Paris Cité, Paris, France; 4 UMR CNRS 8638 COMETE - Chimie Toxicologie Analytique et Cellulaire, Faculté de Pharmacie de Paris, Université Paris Descartes, Sorbonne Paris Cité, Paris, France; 5 Present Address: Laboratoire de bioénergétique fondamentale et appliquée, Université Grenoble Alpes, INSERM U1055, Grenoble, France; 6 Present Address: Biophotonics and Synapse Physiopathology, Laboratoire Aimé Cotton, CNRS, Université Paris-Sud, ENS Cachan, and Université Paris-Saclay, Orsay, France

**Keywords:** Cognitive ageing, Long-term potentiation, Nutrition

## Abstract

The prevalence of cognitive decline is increasing as the ageing population is considerably growing. Restricting this age-associated process has become a challenging public health issue. The age-related increase in oxidative stress plays a major role in cognitive decline, because of its harmful effect on functional plasticity of the brain, such as long-term potentiation (LTP). Here, we show that citrulline (Cit) has powerful antioxidant properties that can limit *ex vivo* oxidative stress-induced LTP impairment in the hippocampus. We also illustrate that a three-month Cit supplementation has a protective effect on LTP in aged rats *in vivo*. The identification of a Cit oxidation byproduct *in vitro* suggests that the antioxidant properties of Cit could result from its own oxidation. Cit supplementation may be a promising preventive nutritional approach to limit age-related cognitive decline.

## Introduction

Ageing is a time-related biological process frequently associated with physical and cognitive decline, possibly leading to pathological states. Age-associated diseases, in particular cognitive disabilities and the associated increase in dependency, have become a public health issue with the ageing of the population^1^. Cognitive decline in physiological ageing is defined by subtle cognitive changes that affect brain structures and functions, such as learning and memory^2,3^. Oxidative stress, *i*.*e*., the imbalance between reactive oxygen species (ROS) production and detoxification, may be an important contributor to the ageing process^4^. Indeed, ageing is associated with oxidative stress resulting from increased ROS production, because of mitochondrial dysfunction and decreased enzymatic and non-enzymatic antioxidant defense systems^5,6^. This results in the accumulation of oxidatively damaged proteins, lipids, and DNA^4^, driving the impairment of various cell functions, such as mitochondrial and lysosomal function^7,8^. The brain is particularly exposed to ROS and is vulnerable to ROS-associated damages, because of its high oxygen consumption and high polyunsaturated fatty acid and low antioxidant enzyme content relative to other organs^9^. The strong association between ageing, oxidative damage in the brain, and cognitive functions suggests that oxidative stress and age-related cognitive decline are closely linked^10^.

Synaptic plasticity, such as long term potentiation (LTP), defined as the long-lasting increase in the efficacy and strength of synaptic transmission of preexisting synapses, has been proposed to be an essential cellular process to encode memory^11^. The hippocampus, a brain structure with a key role in the consolidation of episodic memory, spatial learning, and regulation of emotional behavior, is particularly vulnerable to the ageing process^12^. Hippocampal LTP is impaired in aged individuals, consistent with age-related cognitive deficits^13^. ROS have a physiological role in synaptic plasticity^14^, but elevated oxidative stress may be deleterious. Indeed, enhanced oxidation is associated with impaired hippocampal LTP in aged rats and exposition to high doses (20 µM to 1 mM) of hydrogen peroxide (H2O2) alters LTP expression in hippocampal slices from young rats^10^. Age-related spatial memory deficits in mice are directly correlated with the amount of oxidized proteins in the cortex^15^ and there is a strong correlation between hippocampal oxidative damage and learning impairment in aged rats^16^. Finally, it has also been reported that LOU/C Jall rats, in which oxidative stress does not occur during ageing, display intact hippocampal functional plasticity and memory throughout their lifespan^17,18^. Age-related intracellular oxidative stress (IOS) contributes to the decline in the activation of the N-Methyl-D-Aspartate subtype of glutamate receptors (NMDAR) required for LTP^19^. Glutathione (GSH), a tripeptide present in most cellular compartments, is the major thiol antioxidant and redox buffer of the cell and the GSH status reflects IOS^20^. Importantly, long term treatment with the GSH precursor N-acetyl-cysteine rescues LTP impairment in aged rats^21^. Overexpression of antioxidant enzymes, such as extracellular superoxide dismutase (EC-SOD), protects against age-related LTP and spatial memory impairment^22,23^, consistent with the involvement of ROS toxicity in age-related LTP and cognitive deficits. Moreover, a decrease in the level of endogenous antioxidant compounds, such as α-tocopherol, in the brain impairs LTP in rats^24^.

We focused on a nutritional approach, i.e. the use of citrulline (Cit), an amino acid (intermediate of the urea cycle in mammals) with antioxidant properties^25^, as a potential preventive therapy to minimize age-related cognitive decline. Indeed, Cit is a potent scavenger of hydroxyl radicals (HO°) and can protect cellular enzymes from oxidative damage^26^. Cit can cross the blood-brain barrier^27^ and several studies have shown a beneficial effect of Cit in various neurological diseases associated with oxidative stress, such as transient ischemic stroke^28^. Three-month Cit supplementation in aged rats prevents β-amyloid cleavage in hippocampal rafts, suggesting that it could have a protective effect in Alzheimer’s disease (AD)^29^. However, only a few studies have investigated the antioxidant effect of Cit *in vivo* and its positive effects against age-related disturbances^30,31^, despite its known antioxidant properties described in a chemical system^26^.

The aim of this study is to assess whether Cit prevents oxidative stress and age-related impairment of brain functional plasticity. The Cit protective effect against oxidative stress and the oxidative stress-induced LTP impairment were first assessed (i) *in vitro*, on H2O2-induced IOS and cell death in human neuroblastoma SH-SY5Y cells; this cell model is commonly used to assess neuroprotective effects of compounds against H2O2-induced cell damages and death^32–35^; (ii) *ex vivo*, on H2O2-induced LTP impairment in hippocampal slices from young adult mice, which is a well-defined model of LTP impairment caused by oxidative and H2O2 stress^10,36^. We then studied the effect *in vivo* of a three-month Cit supplementation on LTP in 24 month-old rats where age-related LTP decline has been extensively described^17,18,21,37^. This was completed by the identification of Cit H2O2 oxidation products by reversed-phase ultra-performance liquid chromatography coupled to high resolution mass spectrometry (UPLC-HRMS).

We show that Cit has a protective effect against H2O2-induced oxidative stress and cell death. Cit also protects against H2O2-induced hippocampal LTP impairment. Moreover, we highlight a promising beneficial effect of three months of Cit supplementation *in vivo* on age-related LTP impairment in aged rats. Oxidation of Cit itself may, at least partially, explain the mechanism underlying this protective antioxidant effect.

## Results

### Effect of Cit on a cellular model of H2O2-induced stress

#### Effect of Cit on H2O2-induced IOS

**Figure 1:**
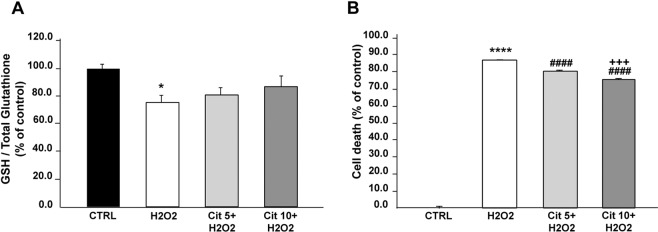
Protective effect of Cit against H2O2-induced oxidative stress in SH-SY5Y cell line. (**A**) Cit effect on intracellular oxidative stress (IOS). IOS was assessed by GSH/Total Glutathione ratio and expressed as % of control. GSH/Total glutathione ratio has been assessed in control conditions (CTRL), after oxidative stress induced by H2O2 500 µM (H2O2), after H2O2 500 µM in the presence of Cit 5 mM (Cit 5 + H2O2) or Cit 10 mM (Cit 10 + H2O2). The significant decrease in GSH/Total glutathione ratio is shown by* (*p < 0.03). (**B**) Cit effect on H2O2-induced cell death. Cell death has been assessed in control condition (CTRL), after treatment with H2O2 500 µM (H2O2) alone and in the presence of Cit 5 mM (Cit 5 + H2O2) or Cit 10 mM (Cit 10 + H2O2). The results are expressed as % of control. The significances are designed as followed: *: H2O2 vs CTRL; #: Cit + H2O2 vs H2O2; +: Cit10 + H2O2 vs Cit5 + H2O2. (^****^p < 0.0001; ^####^p < 0.0001; ^+++^p = 0.0001). Data in A and B are presented as the mean ± SEM of 4 independent experiments.

H2O2 altered IOS, as shown by the significant decrease of approximately 25% of the GSH/Total GSH ratio relative to control (CTRL) (75.2 ± 5.8% *vs* 100.0 ± 3.3% in H2O2 and CTRL, respectively; p < 0.03) (Fig. 1A). Pre-incubation with 5 or 10 mM Cit before H2O2 application led to ratios of 81.0 ± 5.6% and 87.1 ± 8.0%, respectively (Fig. 1A). The ratio observed under Cit + H2O2 conditions was not significantly different from that observed under control conditions (Cit 5 + H2O2 *vs* CTRL: ns; Cit 10 + H2O2 *vs* CTRL: ns).

#### Effect of Cit on H2O2-induced cell death

Although we showed that Cit has a protective effect against IOS under oxidative conditions, it was important to determine whether these effects were associated with a protective effect against cell death. H2O2 led to significant cellular death relative to control conditions (H2O2 *vs* CTRL: p  < 0.0001) (Fig. 1B). Pre-incubation with 5 or 10 mM Cit significantly decreased cell death in a dose-dependent manner (H2O2 *vs* Cit5 + H2O2: p  < 0.0001; *vs* Cit10 + H2O2: p  < 0.0001; Cit5 + H2O2 *vs* Cit10 + H2O2: p = 0.0001) (Fig. 1B).

### Protective effect of Cit on H2O2-induced LTP impairment recorded *ex-vivo* in hippocampal slices from young adult mice

All these experiments were conducted in mice. We wanted to compare the effect of H2O2 (150 µM) on control hippocampal slices or slices pre-treated with Cit (5 mM).

#### Basal synaptic transmission

**Figure 2:**
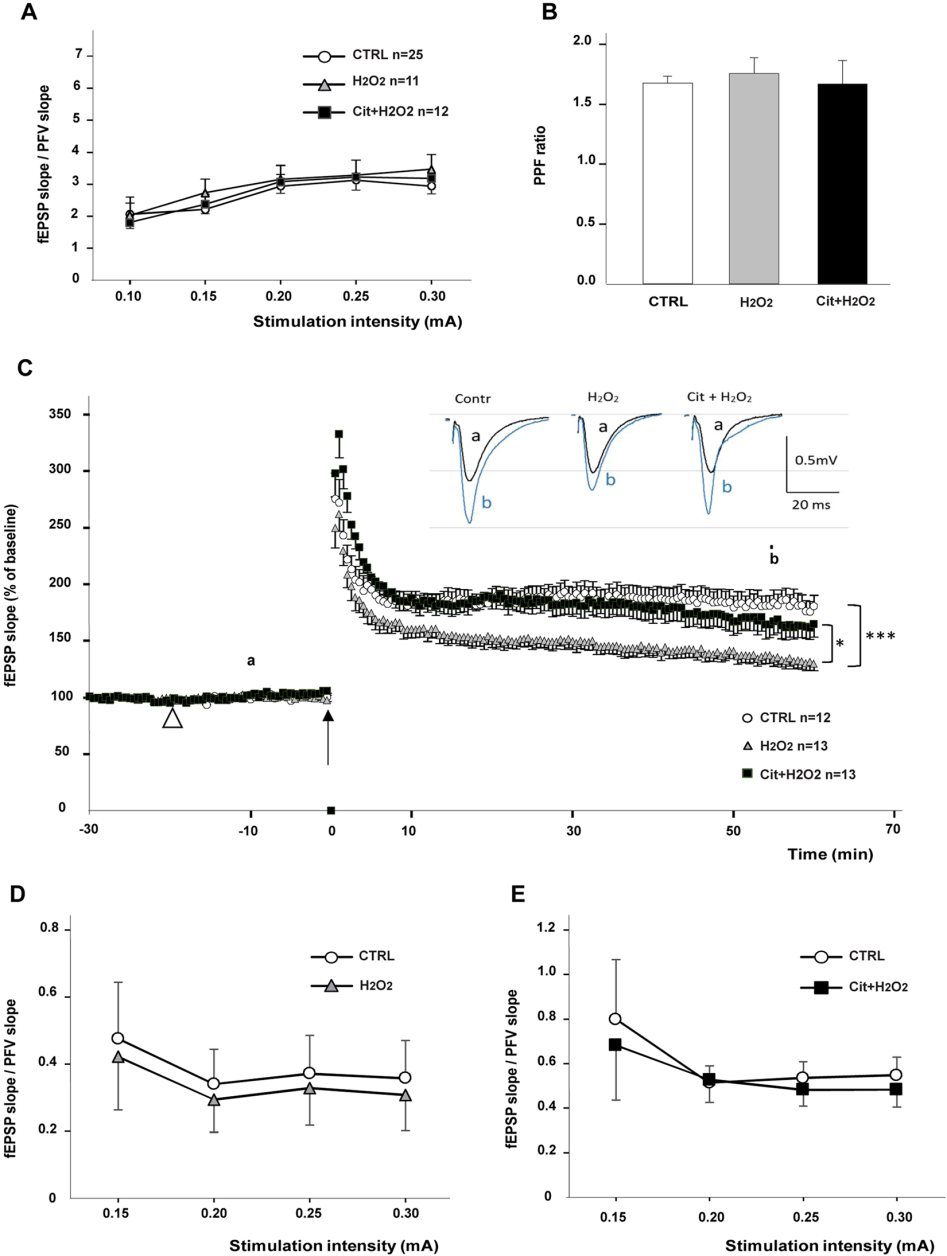
Protective effect of Cit on hippocampal LTP in *ex-vivo* slice preparation of young adult mice. All experiments in Fig. 2 are performed in mice. (**A**) Mean fEPSP/PFV slope ratio of AMPA receptor-mediated synaptic responses plotted against current intensity in control conditions (CTRL, n = 25 slices from 6 mice, white dots) or after application of H2O2 (150 µM, n = 11 slices from 8 mice, grey triangles) or H2O2 (150 µM) in the presence of Cit (5 mM) (n = 12 slices from 4 mice, black squares). (Repeated measures ANOVA, p = 0.91, NS). (**B**) Paired-pulse facilitation (PPF) of synaptic transmission was comparable in the three groups indicating that drug treatments did not alter presynaptic mechanisms underlying glutamate release (p =  0.66, ns, *t*-test). CTRL, n = 27 slices from 9 mice; H2O2, n = 12 slices from 7 mice; Cit + H2O2, n = 7 slices from 5 mice. (**C**) 3 × 100 Hz conditioning stimulus (black arrow) induces a long lasting potentiation of synaptic transmission in the 3 conditions. However, LTP stabilizes to a lower level in the presence of H2O2 (grey triangles) as compared to control conditions (white dots) (F(1,24) = 16.6, ***p < 0.001). This decrease in LTP expression is partially restored in the presence of Cit (black squares) (F(1.25) = 6.9, *p < 0.05). Drugs were applied in the superfusing medium at t = −20 min (white arrowhead) and maintained throughout the experiment. ndividual traces of fEPSPs are shown in the three conditions before (a) and 60 min after stimulation (b). (**D**) Mean fEPSP/PFV slope ratio of NMDA receptor-mediated synaptic responses plotted against current intensity in control conditions (CTRL, n = 10, white dots) and after application of H2O2 (150 µM) (n = 10, grey triangles) (p = 0.9, ns). (**E**) Mean fEPSP/PFV slope ratio of NMDA receptor-mediated synaptic responses plotted against current intensity in control conditions (CTRL, n = 10, white dots) and after application of H2O2 (150 µM) in presence of Cit (5mM) (n = 10, black squares) (p = 0.74, ns).

There was no difference between I/O curves recorded before or after application of H2O2, indicating the absence of a significant effect of H2O2 on basal synaptic transmission mediated by the AMPA subtype of glutamate receptors at this concentration (CTRL: n = 25 slices from 6 mice; H2O2, n = 11 slices from 8 mice) (Fig. 2A). Curves recorded in Cit + H2O2 (n = 12 slices from 4 mice) were not either different (Fig. 2A) (p = 0.91, ns). In addition, these compounds did not significantly change the PPF ratio, indicating that they had no relevant effect on the probability of glutamate release (p = 0.66, ns) (CTRL, n = 27 slices from 9 mice; H2O2, 12 slices from 7 mice; Cit + H2O2, n = 7 from 5 mice) (Fig. 2B).

#### Synaptic plasticity (LTP)

HFS induced a strong LTP of 181.7 ± 9.8% of baseline in CTRL slices (n = 12 slices from 11 mice). After a 20-min incubation with H2O2 (150 µM), the LTP was significantly lower (134.8 ± 6.6% of baseline, n = 13 slices from 10 mice) (F_(1,23)_ = 16.6, p = 0.0004***) (Fig. 2C). The H2O2-induced depression of LTP was partially rescued in slices pre-treated with Cit (165.9 ± 11.2%, n = 13 slices from 9 mice) (F_(1,24)_ = 6.9, p = 0.014*) (Fig. 2C). The magnitude of LTP in CTRL slices and those supplemented with H2O2 + Cit was not statistically different (F_(1,23)_ = 0.8, p = 0.37) (Fig. 2C).

#### Activation of NMDA receptors

The fEPSP-NMDA/PFV ratio obtained for selective NMDA receptor-isolated synaptic potentials was not significantly different in H2O2 (p = 0.9, ns) (Fig. 2D) or H2O2 + Cit (p = 0.74, ns) (Fig. 2E) *vs* CTRL slices, suggesting that the effects of the drugs reported above on LTP were not mainly due to altered activation of NMDA receptors.

### Effect of three-month Cit supplementation on hippocampal LTP in aged rats, a model of “physiological oxidation”

**Figure 3:**
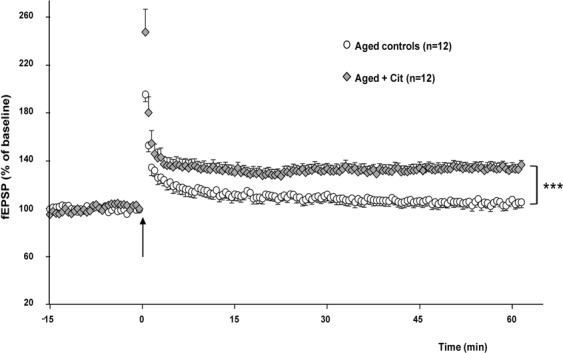
Theta Burst Stimulation-induced LTP is rescued in aged rats fed with Cit. Time course of changes in synaptic efficacy induced by a theta-burst conditioning stimulus (TBS, arrow) applied in the *stratum radiatum* of hippocampal CA1 area in aged control rats and aged rats fed with Cit for 12 weeks. While LTP is not promoted in control animal (white dots, 104.9 ± 4.2%, n = 12), a robust and significant long lasting potentiation is induced in Cit-supplemented animals (133.6 ± 4.7%, n = 12, grey diamonds). The difference in LTP expression between the two groups is statistically significant (F(1,22) = 22; ***p < 0.001).

Experiments were performed in aged rats (24-month old). No significant LTP was recorded in slices from aged rats after theta-burst conditioning stimulation (Fig. 3) as we already demonstrated in previous studies^18,21,37^ (104.9 ± 4.2%, n = 12 slices from 5 rats). Robust LTP was promoted in rats fed with Cit (133.6 ± 4.7%, n = 12 slices from 5 rats), reaching a level generally recorded in younger rats^18,21,37^ (Fig. 3). The difference between the potentiation recorded in aged control and Cit treated rats was statistically significant (F_(1,22)_ = 22, p  < 0.001***).

### UPLC-HRMS analysis of the products formed by the reaction between citrulline and hydroxyl radicals

**Figure 4:**
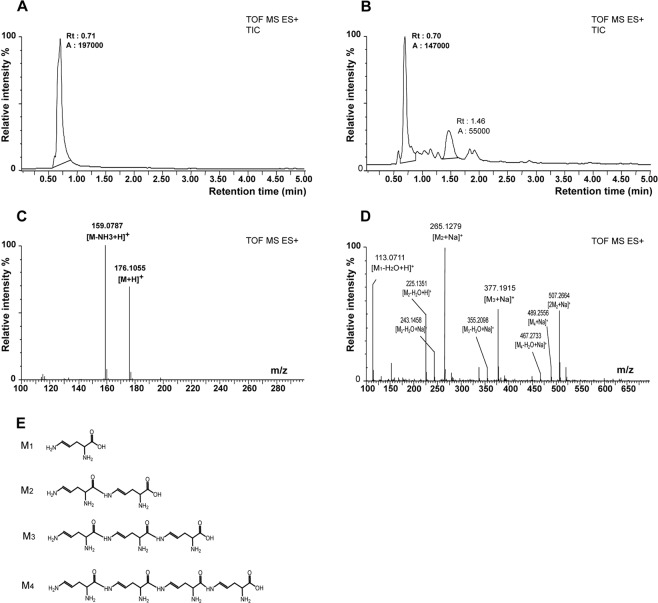
UPLC-HRMS analysis of the products formed *in vitro* by the reaction between Cit and hydroxyl radicals. (**A**) Total ion current chromatograms of Cit 160 mM in H2O. (**B**) Total ion current chromatograms of Cit 160 mM in H2O containing H2O2 90 mM 6 h. Rt: retention time, A: area under curve. (**C**) Mass spectra displays at retention time 0.70 min for Cit 160 mM in H2O. (**D**) Mass spectra displays at retention time 1.46 min for Cit 160 mM in H2O containing H2O2 90 mM 6 h. Annotations were performed following fragmentation experiments on ions m/z 265.1279, 377.1915, 489.2556, 507.2264 and exact mass measurements (<5 ppm). (**E**) M_n_ correspond to condensation products of M1 formed in ion source of the mass spectrometer.

We analyzed Cit and Cit + H2O2 samples by UPLC-HRMS. Figure 4A shows the total ion current chromatogram of the Cit sample with a single peak at a retention time (Rt) of 0.71 min. MS signals of 176.1055 and 159.0787 *m/z*, corresponding to [M + H]^+^ and [M +H-NH_3_]^+^ ions, respectively, confirmed the identity of this peak as Cit (Fig. 4C). The total ion current chromatogram of the Cit + H2O2 sample displayed several peaks corresponding to potential products resulting from the reaction between Cit and H2O2, in addition to the peak of Cit at 0.71 min mentioned above (Fig. 4B). We examined the peak with the largest area under the curve, corresponding to the peak at Rt 1.46 min (Fig. 4D), to identify the MS signal of the main product of the reaction. The mass spectrum at Rt 1.46 min displayed *m/z* signals of 153.0639 (M1), 265.1279 (M2), 377.1915 (M3), and 489.2664 (M4), separated by 112 Th increments, as mentioned by Akashi *et al*.^26^, suggesting a condensation or polymerization reaction of the initial product (M1).

These peaks correspond to a series of [M + Na]^+^ species with M being the reaction product M1 or its auto-condensation derivatives, M2-M4. This attribution was confirmed by another series of [M + H-H_2_O]^+^ peaks at *m/z* of 113.0711, 225.1351, 337.1992, and 449.2629 and by series of [M + Na-H_2_O]^+^ peaks at *m/z* of 243.1458, 355.2098, and 467.2733. Exact mass measurements of these peaks suggest the elemental formula C5H10N2O2 for the M1 compound. This corresponds to the putative chemical structures shown in the scheme (Fig. 4E). M_1_ corresponds to Cit in which the ureido group has been replaced by an amino group, suggesting the importance of the long side chain of Cit for this reaction.

## Discussion

It is widely accepted that tissue and cellular oxidative damage, due to increased ROS production and decreased antioxidative defenses, are closely associated with ageing^5^. This age-related oxidative damage impairs many physiological processes, in particular synaptic plasticity of the brain, leading to age-related cognitive decline^12^. Age-related alterations of LTP correlate with oxidative cellular damage and *ex-vivo* experiments have shown that oxidative stress has a detrimental effect on this synaptic process^21^. Discovery of the antioxidant and nitric oxide modulating properties of Cit has generated renewed interest in this amino acid in several fields of medicine^38,39^ highlighting, among others, its beneficial effects in neurological insult, such as ischemic stroke, in which oxidative stress plays a crucial role^28^.

Our study assessed the ability of Cit to limit or mitigate the harmful effects of ageing on the capacity of neuronal networks to express functional plasticity, such as LTP. We first assessed the antioxidative properties of Cit in SH-SY5Y cells subjected to exogenous H2O2-induced stress, as increased oxidative stress plays a crucial role in age-related cognitive loss and LTP impairment. We studied the effect of Cit on IOS, as age-related IOS is involved in LTP impairment^19^. H2O2 stress significantly impaired IOS, as expected, and pre-incubation with Cit restored IOS similar to control conditions. In addition to its protective effect on IOS, pre-incubation with Cit led to a significant decrease in H2O2-induced cell death in a dose-dependent manner. Demonstrating the antioxidant properties of Cit in a biological system was crucial, because this has been little studied. Indeed, only two studies have shown the biological antioxidant effects of Cit: (1) oral-Cit supplementation has a protective effect against brain protein carbonylation^30^ and (2) three months of oral-Cit supplementation reduces serum and lipoprotein susceptibility to oxidation in aged rats^31^.

We studied LTP in hippocampal slices from mice subjected to H2O2 stress that were pretreated with Cit, or not, to assess whether the protective biological effect of Cit against exogenous oxidative stress is able to limit the impairment of functional plasticity at synapses of the hippocampus. Electrophysiological recordings showed that Cit partially rescued the H2O2-induced LTP deficit. The H2O2-induced LTP deficit was not associated with altered basal synaptic transmission or glutamate release. Thus, the protective effect of Cit does not appear to be mediated by an action on NMDA receptors, which are normally critical for LTP. The mechanism by which acute exogenous H2O2 stress alters LTP appears to be different from that associated with ageing. Indeed, age-related LTP impairment is clearly associated with altered NMDAR activation, which is tightly associated with IOS^19^. This observed difference may be related to the fact that oxidative stress in physiological ageing is less intense than acute H2O2-induced oxidative stress and spread out over a much longer period. The exact targets necessary for LTP that are altered by exogenous H2O2 and restored by Cit are yet to be determined. Nonetheless, our results highlight the protective effect of Cit against oxidative-induced toxicity. It was important to confirm the protective effect of Cit *in vivo* using the validated model of age-related physiological neuron oxidation^21,40^, because of the differences between the ageing process and acutely-induced exogenous oxidative stress. It has been demonstrated in middle-aged rats, the emergence of episodic memory alterations associated with a redox-sensitive decline in NMDAR function (Lee *et al*. 2012; Kumar & Foster 2013). We showed here that the impaired NMDAR-dependent LTP recorded in the hippocampus of aged rats displaying age-related oxidative damage was rescued by Cit supplementation, thus confirming the protective effect of Cit in the context of physiological ageing.

These results led us to examine how Cit protects the brain from oxidative stress. It was recently demonstrated that the antioxidant properties of Cit are not related to the modification of mitochondrial oxygen-free radical production^41^. We suggest that Cit can neutralize HO° produced from H2O2 through Fenton’s reaction^26^. Indeed, we show that Cit can be oxidized by H2O2 (more precisely, by HO°), in the presence of iron ions, to form oxidation products. The auto-condensation derivatives M2-M4 were detected in the mass spectrum of products with an at Rt of 1.46 min. The same Rt for all three derivatives suggests that they were formed during mass spectrometry analysis, and thus do not exist in solution. The auto-condensation reaction varied with ion source conditions (data not shown), in accordance with this hypothesis. Thus, the peak for M1 at *m/z* 113.0711, corresponding to [M1-H2O+H]^+^, is likely the main oxidation product of Cit. Our results show the main oxidation product, M1, to be smaller than Cit, in contrast to Akashi *et al*., who suggested that the oxidation products of Cit are larger in molecular weight than Cit. The protective mechanism of Cit appears to have certain similarities with α-tocopherol, as Cit acts as a “suicide substrate” to neutralize ROS^42^. However, it is unlikely that Cit can be reformed from its oxidized product, in contrast to α-tocopheryl (oxidized form of vitamin E) which can be reduced by ascorbic acid to α-tocopherol.

Although we are the first to demonstrate the protective effect of Cit on the expression of functional plasticity in the ageing hippocampus, other studies have already assessed the ability of antioxidant compounds to protect against exogenous oxidative insults or age-related alterations in synaptic plasticity: tanshinone IIA, a component of Salvia miltiorrhiza Bunge used in Chinese medicine, has antioxidative properties that protect against *ex vivo* H2O2-induced neuronal cell death and LTP impairment^43^. Daily intraperitoneal injection of this compound for 30 days was also able to rescue impaired hippocampal LTP and reduce memory impairment in the six-month old APP/PS1 mouse model of AD (Li *et al*., 2016). In addition, aged rats that received a diet supplemented with α-tocopherol for three months sustained LTP that was indistinguishable from that of young rats^44^. However, observational studies provided only modest evidence of a beneficial impact of α-tocopherol consumption on cognitive impairment and data from randomized clinical trials have failed to provide convincing evidence for clinically-relevant effects of vitamin E, either in delaying cognitive decline in aged non-demented adults or preventing or limiting progression of dementia in mild cognitive impairment or AD patients, respectively^45^. A recent study of our group showed that 12-months of N-acetyl-L-cysteine (L-NAC) supplementation restored LTP induction in aged rats^21^. Several randomized clinical trials of L-NAC supplementation suggest that it may provide a clinical benefit by reducing cognitive changes in oxidative stress-associated disorders, such as AD, but very few studies have examined its impact on specific age-related cognitive decline^46^. In general, the differences between the studies (dose, treatment duration, sample size, design of the study) make the results difficult to interpret. Thus, despite the promising nature of preclinical data obtained for certain antioxidant compounds, no clinical trials have yet been able to provide convincing evidence of a beneficial effect of these drugs on age-related cognitive decline. In addition, recent data highlight that consumption of well-known antioxidants, such as vitamin E or beta-carotene, combined with a balanced diet, may be harmful for health^47^. For example, long-term supplementation with vitamin E can significantly increase the risk of prostate cancer^48^. Available data on the toxicity of Cit suggest that Cit is safe. Indeed, long-term Cit supplementation in animals^31^, as well as supplementation for several weeks in humans, are well-tolerated and give no major side effects^49–51^. Cit may thus be a promising nutritional approach to efficiently counteract, or limit, age-related cognitive decline in the ageing population.

Although this study highlights Cit supplementation as a promising nutritional approach to limit age-related LTP decline, we are faced with two main limitations.

First although the specific *in vitro*, *ex vitro* and *in vivo* models used here are well-validated to answer the study questions, their diversity may limit extrapolation from one model to the other.

However, studies show that (i) neuroprotective mechanisms against H2O2 stress are identical between SH-SY5Y cells and mice hippocampal neurons^52^; and (ii) mechanisms of LTP in area CA1 described in multiple reviews (see for instance Sweatt, 2016^53^) are comparable in mice and rats; therefore we can reasonably assume that our results might presumably be extended from one model to the other.

Secondly, our experiments did not permit to go further and evaluate whether Cit supplementation can reverse any potential cognitive deficits. Indeed, assessment of cognitive function in old Sprague-Dawley rats is particularly complicated because of frequent age-related physical impairments such as hindlimb neuromuscular disturbance^54^ or retinal degeneration^55^. Further experiments, on certified models of cognitive decline with age, are needed.

## Conclusion

The results of this study strongly support the potential of Cit supplementation to mitigate the impairment of synaptic plasticity in neuronal networks of the ageing brain. The powerful antioxidant properties of Cit may explain, at least in part, the protective effect of Cit supplementation on age-related LTP impairment. In addition, no adverse side effects have been reported, making it possible to start Cit supplementation in midlife, before age-related damage occurs. Although further studies are needed to confirm our results, Cit appears to be a promising preventive nutritional approach to limit age-related impairment of functional plasticity in the ageing brain and thus cognitive decline.

## Materials and Methods

### Animals and nutrition intervention design

6-week old male C57/Bl6 mice (n = 11) were purchased from Janvier Labs (Le Genest Saint Isle, France).

21-month old male Sprague-Dawley rats (aged rats, n = 10) were purchased from Charles River (L′Arbresle, France) and housed in groups of 2–3 per cage during a 12-week nutrition intervention.

Rats were fed *ad libitum* with a standard diet (17% protein, 3% fat, 59% carbohydrate, and 21% water plus fibers, vitamins, and minerals; A04, Safe, France) for a 1-week equilibration period in which food intakes were recorded. Main food intake was 24 g/d.

After acclimatization, the rats were randomized in 2 groups: «aged control» (n = 5) and « aged + Cit » (n = 5). In «aged + Cit» group, rats ingested *ad libitum* for 12 weeks a standard diet supplemented with Cit (13.5 g N/ kg of standard diet) providing 1 g of Cit/kg of body weight/day. The «aged control» rats were fed *ad libitum* with the standard diet. No significant differences in the amount of Cit ingested per day was found among rats in the «aged + Cit» group.

The dose of Cit provided in the diet (1 g/kg/day) was extrapolated from the optimal dose determined in humans^51^. Numerous studies showed that this dose was effective in rats^56,57^ and especially in older rats^31^.

### Ethics statements

All animal procedures were carried out in compliance with French regulations and in strict accordance with the recommendations of the European Economic Community (63/2010) and approved by the regional animal ethics committee (Comité Régional d′Ethique pour l′Expérimentation Animale Ile-de-France) under authorization no. P2.CM.058.08.

### Chemicals and reagents

Citrulline was a gift from the CITRAGE company (Creteil, France). H2O2 and Ethylenediaminetetraacetic acid iron (III) sodium salt was purchased from Sigma-Aldrich (St. Louis, MO, USA). Dulbecco’s modified Eagle’s medium (DMEM), high glucose, glutamine (GlutaMax^TM^), fetal calf serum (FCS), Penicillin-streptomycin (pen-strep), and N-2 supplement for neuronal cell culture were purchased from Gibco Invitrogen (Carlsbad, CA, USA).

### Cit treatment and oxidative stress protocol in the SH-SY5Y cell line

Human neuroblastoma SH-SY5Y cells were cultured in DMEM GlutaMax, 10% FCS, and 0.1% pen/strep for 12 h, and the culture medium was changed to DMEM GlutaMax^TM^, 1% N-2 supplement, 0.1% pen/strep, with or without Cit (5 mM or 10 mM), before a 5-h pre-incubation period. H2O2 was then added to the culture medium at 250 µM and the cultures incubated for 1 h, or at 500 µM and the cultures incubated for 2 h, for the assessment of IOS and cell death, respectively.

### Assessment of intracellular oxidative stress (IOS)

Reduced GSH and total glutathione were measured with the GSH/GSSG Ratio Detection Assay Kit II (Fluorometric-Green) from Abcam (ab205811, Cambridge, UK) according to the manufacturer’s instructions. Upon reacting with GSH the dye used in this assay becomes fluorescent. Before analysis, wells were washed with 1X PBS. Four independent experiments were performed. The GSH/Total GSH ratio is expressed as the mean ± SEM (percentage of control condition).

### Assessment of cell death

Cell death was assessed using the Cell Titer-Glo-Luminescent Cell Viability kit (Promega, Charbonnières, France) according to the manufacturer’s instructions. The quantification of intracellular ATP, that reflects metabolically active cells, was used to measure cell death. Four independent experiments were performed. Cell death is expressed as the mean ± SEM (percentage of control condition).

### UPLC-HRMS analysis

A “Cit sample”, containing only 160 mM Cit in H2O, and a “Cit + H2O2 sample”, containing 160 mM citrulline, 88 mM H2O2, and 0.1 mM EDTA-Na-Fe(III), were prepared and incubated for 6 h at 25 °C, as described by Akashi (Akashi *et al*., 2001). Thereafter, the samples were stored at −80 °C until UPLC-HRMS analysis. Analysis was performed using reversed-phase ultra-performance liquid chromatography (RP-UPLC) coupled to a hybrid quadrupole-orthogonal time-of-flight mass spectrometer equipped with an electrospray ionization (ESI) source (ACQUITY UPLC^®^ and SYNAPT^®^ G2 High Definition MS™ mass spectrometer, Waters, Manchester, UK). Analyses were achieved using a CSH^®^ C18 1.7 μm column (2.1 × 100 mm) maintained at 40 °C. Data were collected in positive (ESI+) ion mode. ESI source parameters were as follows: source temperature 120 °C, desolvation temperature 550 °C, cone gas flow 20 L.h^−1^, desolvation gas flow 1000 L.h^−1^, capillary voltage for ESI+ ion mode 3,000 V. Centroid mass corrected spectra were acquired over the 50–1000 *m/z* range with a scan time of 0.1 s and an interscan delay of 0.01 s using a target mass resolution of 21,500 (FWHM as defined at 500 *m/z*). Mass measurements were corrected during acquisition using a solution of leucine enkephalin as an external reference (Lock-Spray™).

### Neurotransmission and synaptic plasticity in the CA1 area of hippocampal slices from young adult mice and aged rats

For pharmacological experiments in mice, H2O2 (150 μM), Cit (5 mM), or both were added to the aCSF 20 min before and during the establishment of the baseline and maintained throughout recording. Preliminary experiments showed that 150 μM H2O2 had a markedly harmful effect on LTP under our experimental conditions. The lowest Cit concentration tested in this study able to counteract the harmful effect of H2O2 in the SH-SY5Y cell line was 5 mM.

#### *Ex vivo* slice preparation

Mice or rats were anesthetized with isoflurane and decapitated. The brain was rapidly removed from the skull and placed in ice-cold (0–3 °C) artificial cerebrospinal fluid (aCSF) containing the following: 124 mM NaCl, 3.5 mM KCl, 1.5 mM MgSO4, 2.5 mM CaCl2, 26.2 mM NaHCO3, 1.2 mM NaH2PO4, and 11 mM glucose. Hippocampal sections (400 µm thick) were cut using a tissue chopper, then placed in the aCSF solution and maintained at 27 °C for at least 1 h before recording. Each slice was individually transferred to a submersion-type recording chamber and continuously superfused with aCSF medium equilibrated with 95% O2, 5% CO2.

#### Recordings

Extracellular recordings were obtained at room temperature from the apical dendritic layer of the CA1 area using micropipettes filled with 2 M NaCl. Presynaptic fiber volleys (PFVs) and field excitatory postsynaptic potentials (fEPSPs) were evoked by electrical stimulation of Schaffer collaterals and commissural fibers located in the *stratum radiatum*.

#### Synaptic transmission

Input/output (I/O) curves were constructed to assess the responsiveness of the AMPA subtype of glutamate receptors to electrical stimulation to compare the effects of Cit and H2O2 on basal synaptic transmission. The slopes of three averaged PFVs and fEPSPs were measured and the fEPSP/PFV ratio plotted against the stimulus intensity. I/O curves were thus constructed before and in the presence of Cit and H2O2.

Paired-pulse facilitation (PPF) of synaptic transmission, an electrophysiological paradigm that investigates presynaptic release of transmitter, was induced by electrical stimulation of Schaffer collaterals/commissural fibers with paired pulses at an inter-stimulus interval of 40 ms. The PPF was quantified as the ratio of the second fEPSP slope over that of the first response.

#### Synaptic plasticity

A test stimulus was applied every 10 s in control medium and adjusted to get a fEPSP with a baseline slope of 0.1 V/s to investigate LTP. The averaged slope of three fEPSPs was measured for 15 min before high frequency stimulation (HFS, 3 × 100 Hz, separated by 20 s) in mice or theta burst stimulation (five trains of four pulses at 100 Hz, separated by 200 ms) in aged rats. Testing with a single pulse was then resumed for 60 min to determine the level of LTP. Drugs, H2O2 or Cit + H2O2, were applied during 20 min, after a 10 min baseline.

NMDA receptor-mediated fEPSPs were investigated by perfusing slices with low-Mg^2+^ (0.1 mM) aCSF supplemented with the AMPA/kainate receptor antagonist 2,3-dioxo-6-nitro-1,2,3,4-tetrahydrobenzoquinoxaline-7-sulfonamide (NBQX, 10 µM) for 20 min.

### Statistical analysis

For cell culture experiments, data are expressed as the mean ± SEM. Statistical analyses were performed using parametric ANOVA coupled with Tukey’s post-hoc test. The significance was confirmed by non-parametric ANOVA (Kruskal-Wallis). Significance was set at *P* ≤ 0.05.

The significance of LTP expression was determined by comparing the 15 min of baseline recordings with values recorded between 45 and 60 min after the conditioning stimulation. The significance of changes in LTP magnitude between groups and/or drugs was determined by comparing the last 15 min of recordings. *P* values were calculated using multivariate analysis of variance followed by Tukey’s *post hoc* tests (StatView software) to account for the correlations inherent to repeated measures in electrophysiological recordings.

## Data Availability

All data generated or analysed during this study are included in this published article.
